# Differential Impact of EGFR-Targeted Therapies on Hypoxia Responses: Implications for Treatment Sensitivity in Triple-Negative Metastatic Breast Cancer

**DOI:** 10.1371/journal.pone.0025080

**Published:** 2011-09-22

**Authors:** Abderrahim El Guerrab, Rabah Zegrour, Carine-Christiane Nemlin, Flavie Vigier, Anne Cayre, Frederique Penault-Llorca, Fabrice Rossignol, Yves-Jean Bignon

**Affiliations:** 1 Department of Oncogenetic, Centre Jean Perrin, Clermont-Ferrand, France; 2 ADELBIO, Faculty of Medicine, Centre Biomédical de Recherche et Valorisation, Clermont-Ferrand, France; 3 Department of Pathology, Centre Jean Perrin, Clermont-Ferrand, France; Virginia Commonwealth University, United States of America

## Abstract

**Background:**

In solid tumors, such as breast cancer, cells are exposed to hypoxia. Cancer cells adapt their metabolism by activating hypoxia-inducible factors (HIFs) that promote the transcription of genes involved in processes such as cell survival, drug resistance and metastasis. HIF-1 is also induced in an oxygen-independent manner through the activation of epidermal growth factor receptor tyrosine kinase (EGFR-TK). Triple-negative breast cancer (TNBC) is a subtype of invasive breast cancer characterized by negative expression of hormonal and HER2 receptors, and this subtype generally overexpresses EGFR. Sensitivity to three EGFR inhibitors (cetuximab, gefitinib and lapatinib, an HER2/EGFR-TK inhibitor) was evaluated in a metastatic TNBC cell model (MDA-MB-231), and the impact of these drugs on the activity and stability of HIF was assessed.

**Methodology/Principal Findings:**

MDA-MB-231 cells were genetically modified to stably express an enhanced green fluorescent protein (EGFP) induced by hypoxia; the Ca9-GFP cell model reports HIF activity, whereas GFP-P564 reports HIF stability. The reporter signal was monitored by flow cytometry. HIF-1 DNA-binding activity, cell migration and viability were also evaluated in response to EGFR inhibitors. Cell fluorescence signals strongly increased under hypoxic conditions (> 30-fold). Cetuximab and lapatinib did not affect the signal induced by hypoxia, whereas gefitinib sharply reduced its intensity in both cell models and also diminished HIF-1 alpha levels and HIF-1 DNA-binding activity in MDA-MB-231 cells. This gefitinib feature was associated with its ability to inhibit MDA-MB-231 cell migration and to induce cell mortality in a dose-dependent manner. Cetuximab and lapatinib had no effect on cell migration or cell viability.

**Conclusion:**

Resistance to cetuximab and lapatinib and sensitivity to gefitinib were associated with their ability to modulate HIF activity and stability. In conclusion, downregulation of HIF-1 through EGFR signaling seems to be required for the induction of a positive response to EGFR-targeted therapies in TNBC.

## Introduction

Recently, breast carcinomas have been classified into the following clinicopathological subtypes based on molecular profiling: luminal, human epidermal growth factor receptor (HER2) overexpressing, normal-like, and basal-like breast cancers (BLBCs) [1]. BLBCs express basal markers such as cytokeratins and epidermal growth factor receptor (EGFR) and are generally negative for HER2 expression and both the progesterone and estrogen receptors [2], [3], [4]. This breast cancer subtype is also called triple-negative breast cancer (TNBC) and represents about 15% of invasive breast carcinomas. TNBC does not respond to hormonal therapy (such as tamoxifen or aromatase inhibitors) or HER2-targeted therapies such as Herceptin (trastuzumab). TNBC shows an aggressive pattern of progression with a high rate of early-occurring metastasis [5]. TNBC is one of the most challenging subtypes of invasive breast cancer to treat because of the lack of specific therapies. However, as mentioned previously, EGFR expression is seen in a majority of TNBC cases, thus providing a potential targeted therapy [6].

EGFR is a tyrosine kinase receptor that triggers the phosphatidylinositol 3-kinase (PI3K)/Akt pathway upon activation [7]. In many human cancers, such as colorectal cancer and non-small-cell lung cancer, EGFR overexpression is correlated with cellular proliferation, angiogenesis and tumor growth, leading to disease progression involving invasion and metastasis [8]. Epidermal growth factor (EGF) has been shown to stimulate the migration of breast [9], prostate [10] and renal carcinoma cells [11]. Clinical studies have shown that patients with brain metastasis are prone to have primary tumors that are hormone receptor negative and overexpress HER2 and/or EGFR [12]. Furthermore, EGF can promote the migration of a TNBC cell line (MDA-MB-231) through the PI3K/Akt pathway, suggesting that EGF may be involved in breast cancer progression [13].

In recent years, several EGFR inhibitors have been developed to treat advanced cancers by disrupting PI3K/Akt signaling cascades and circumventing the development of metastasis [14]. Different approaches have been used to target EGFR, including small molecules such as ZD1839/gefitinib (Iressa) or GW572016/lapatinib (Tyverb) and humanized monoclonal antibodies such as cetuximab (Erbitux). Gefitinib is a selective EGFR tyrosine kinase inhibitor, and lapatinib is a dual inhibitor of EGFR and HER2 tyrosine kinase activity. However, evidence of resistance to these drugs has been described, with hypoxia in solid tumors being a putative causative factor. Hypoxic tumors are characterized by more aggressive and metastatic phenotypes, with lower sensitivity to treatments, leading to poor prognosis [15], [16].

Oxygen homoeostasis plays a central role in the development and growth of tissues. In solid tumors, cells within the tumor have to adapt their metabolism to the low availability of oxygen by increasing the expression of genes involved in processes such as angiogenesis, erythropoiesis, glucose metabolism, cell survival and cell proliferation. Gene transcription is regulated by a transcription factor called hypoxia-inducible factor 1 (HIF-1) [17], [18]. The HIF-1 protein is heterodimeric, composed of an alpha subunit and a beta subunit that are constitutively expressed under normoxia. However, HIF-1 alpha is rapidly degraded and cannot be detected in cells [19]. HIF-2 alpha is another isoform of the alpha subunit with similar structure. HIF-1 alpha was the first isoform identified and is ubiquitously expressed [20]. HIF-1 alpha is targeted for degradation through the hydroxylation of its oxygen-dependent degradation domain (ODDD) by 2-oxoglutarate-dependent dioxygenases, which are prolyl hydroxylases (PHDs). These enzymes use oxygen as a cofactor and hydroxylate two conserved proline residues (Pro402 and Pro564) in the ODDD [21]. Prolyl-hydroxylated HIF-1 alpha is recognized by the Von Hippel-Lindau protein (pVHL), which is a component of an E3 ubiquitin ligase complex that drives polyubiquitylation and proteasomal degradation [22], [23]. In addition, an asparagyl hydroxylase called factor inhibiting HIF-1 (FIH-1) hydroxylates asparagine 803 in the C-terminal transactivation domain (CAD), thus preventing HIF-1 alpha from interacting with transcriptional coactivators such as p300 [24]. Under hypoxic conditions, HIF-1 alpha is stabilized and accumulates because of the inactivation of dioxygenases. HIF-1 alpha is translocated to the nucleus where it associates with HIF-1 beta. HIF-1 binds to hypoxia-responsive elements (HRE), which are specific sequences (5′-RCGTG-3′) in the promoter regions of target genes, allowing transcriptional activation [25].

The PI3K/Akt signaling cascade also regulates HIF-1 alpha protein levels in an oxygen-independent manner via the mammalian target of rapamycin (mTOR) [26]. In this way, HIF-1 alpha synthesis is upregulated in response to EGFR activation [27], [28]. Anti-EGFR-targeted therapies may decrease the hypoxia-induced accumulation of HIF-1, leading to a reduction in HIF-1-mediated drug resistance and metastasis through the inhibition of EGFR signaling. In this study, the effects of three EGFR inhibitors (gefitinib, lapatinib and cetuximab) on the stability and activity of hypoxia-induced factors have been evaluated in a metastatic basal-like TNBC model (MDA-MB-231). We also assessed the effects of these drugs on cell migration and cell viability.

## Results

### Stable fluorescent mammary cell lines for examining responses to hypoxia

**Figure 1:**
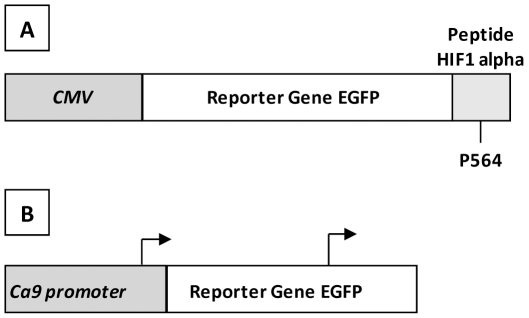
Diagrams depicting the GFP-P564 and Ca9-GFP molecular construct. (A) The *GFP-P564* construct encodes a fusion protein in which EGFP is fused to a peptide containing the Pro564 residue of the HIF alpha subunit. This residue is hydroxylated under normoxic conditions, allowing it to be recognized by pVHL and degraded by the proteasome pathway. Under hypoxic conditions, the peptide is not degraded, and the EGFP fusion protein is continuously expressed under the control of the CMV promoter. This construct reports the post-translational stability of HIF alpha subunits. (B) In the Ca9-GFP cell model, EGFP is activated by HIF dimers under hypoxic conditions. HIF complexes bind to the *Ca9* promoter resulting in EGFP expression, which can be detected by flow cytometry. Under normoxic conditions, HIF alpha subunits are continuously degraded, and Ca9-GFP cells do not fluoresce. This model reports the transcriptional activity of HIF complexes.

**Figure 2:**
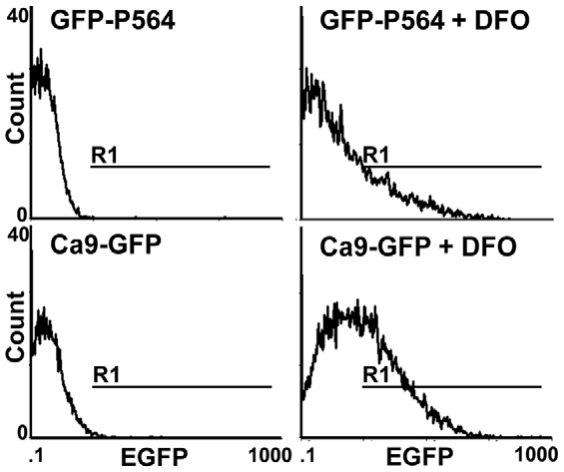
Flow cytometric analysis of EGFP fluorescence in GFP-P564 and Ca9-GFP cells. Cells were treated with 250 µM desferioxamine (DFO) for 24 H or left untreated. R1 is the region of fluorescence induced by DFO. The percentage of cells in R1 and the mean fluorescence increased in response to hypoxic conditions.

To estimate the transcriptional activity of HIF complexes and the stability of HIF alpha isoforms, two EGFP-labeled cell lines were engineered from the metastatic TNBC cell line MDA-MB-231. A clonal population of MDA-MB-231 cells was transfected with one of two plasmids coding for EGFP. The fluorescent signal of the cells was activated by hypoxia through the *Ca9* promoter in the Ca9-GFP cell model and via the stability of EGFP in the GFP-P564 cell model (Fig. 1). The transfected cell populations were characterized by fluorometry, flow cytometry and microscopy. The most homogenous clones with the highest fluorescence intensity were selected. Stability was assessed by measuring fluorescence after various numbers of passages and freeze-thaw cycles and the removal of antibiotic selection pressure. Fluorescent proteins were chosen for these models as, unlike luminescent systems using luciferase, they do not consume molecular oxygen, and therefore, the induction process is unbiased and more physiologically relevant. Cellular phenotypes were determined by flow cytometry. Forward and side scatter signals were used to limit the analysis to viable cells. Cell counts (y axis) were plotted against the EGFP signal (x axis). EGFP-negative cells were used to set the photomultiplicator (PM) amplification and background signals (Fig. 2).

**Figure 3:**
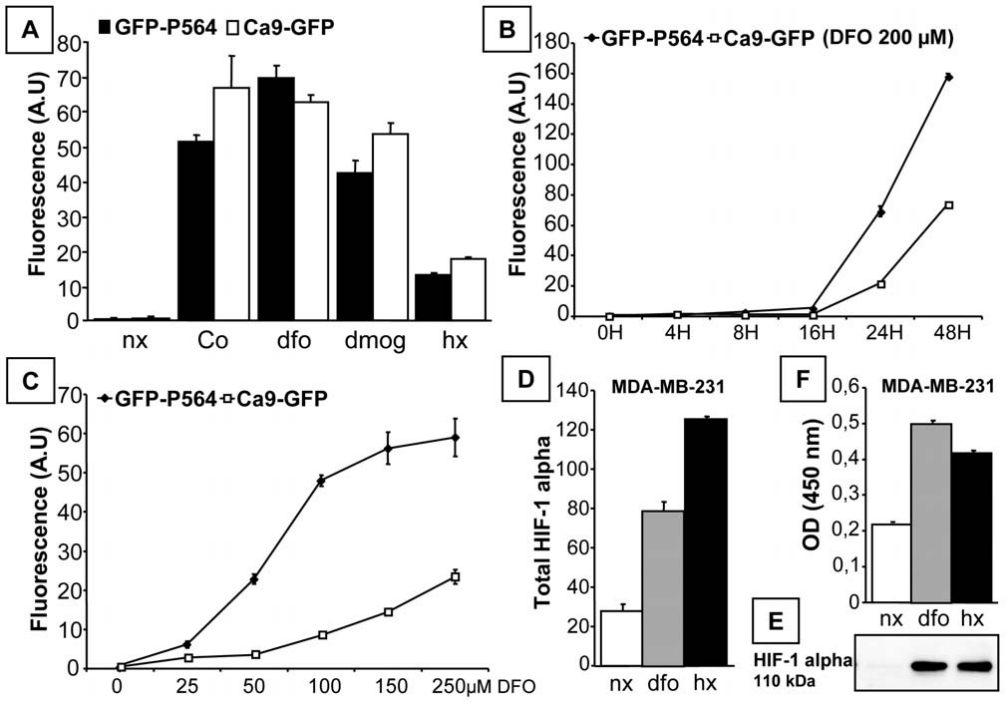
Validation of the GFP-P564 and Ca9-GFP hypoxia-inducible reporters. Hypoxic stimuli induced HIF-1 alpha accumulation in MDA-MB-231 cells and increased fluorescence in the GFP-P564 and Ca9-GFP cell models. Cells were treated with 200 µM cobalt (Co), 200 µM DFO, or 1 mM dimethyloxalylglycine (DMOG) or exposed to 1% O_2_ for 24 H (hx). Untreated cells were incubated under normoxic conditions (nx) and used as controls. (A) Fluorescence was evaluated by flow cytometry, and the signal significantly increased (p<0.05) under hypoxic conditions. DFO induced (B) time- and (C) dose-dependent fluorescence, as assessed by flow cytometry in GFP-P564 and Ca9-GFP cells. (D) Total HIF-1 alpha was quantified in whole MDA-MB-231 cells using an ELISA assay. (E) HIF-1 alpha protein levels in MDA-MB-231 cells were analyzed by western blot. (F) HIF-1 alpha DNA-binding activity was analyzed in MDA-MB-231 nuclear extracts using a 96-well ELISA assay; the absorbance was assessed at 450 nm. Each well was coated with a dsDNA sequence containing the HIF-1 alpha response element. The data represent three independent experiments (average mean ± SEM).

EGFP fluorescence in the Ca9-GFP and GFP-P564 models strongly increased in the presence of hypoxia-mimicking reagents (Co, DFO and DMOG) and under hypoxia (1% O_2_). In both cell models, induction was stronger in the presence of the mimetic compounds compared with hypoxia. EGFP was induced up to 40-fold in GFP-P564 cells treated with 200 µM DFO for 24 H and 35-fold in Ca9-GFP cells (Fig. 3A). As shown in time-course experiments (Fig. 3B), induction in response to 200 µM DFO was detected at a significant level only after 20 H. Treatment with DFO also induced a dose-dependent increase in cellular fluorescence (Fig. 3C). This was correlated with the endogenous level of HIF-1 alpha protein in MDA-MB-231 cells. HIF-1 alpha levels, measured by whole-cell ELISA, significantly increased under DFO and hypoxic conditions (Fig. 3D). Furthermore, HIF-1 alpha expression and DNA-binding activity, as assessed in nuclear extracts, were also induced by DFO and hypoxia (Fig. 3E-F). Taken together, these data demonstrate the stability of the selected reporter clones in terms of their capacity for induction by hypoxia and mimetic compounds targeting dioxygenases, which have been implicated in the regulation of hypoxia responses. Time- and dose-dependent effects were also observed. The induction of EGFP followed HIF-1 alpha protein expression and consensus HRE DNA binding. The Ca9-GFP and GFP-P564 cell lines constituted attractive models of metastatic basal-like TNBC in which to study hypoxia signaling during *in vitro* drug assays.

### Effect of EGFR-targeted therapies on the hypoxia-induced stability and activity of HIF-1 alpha

**Figure 4:**
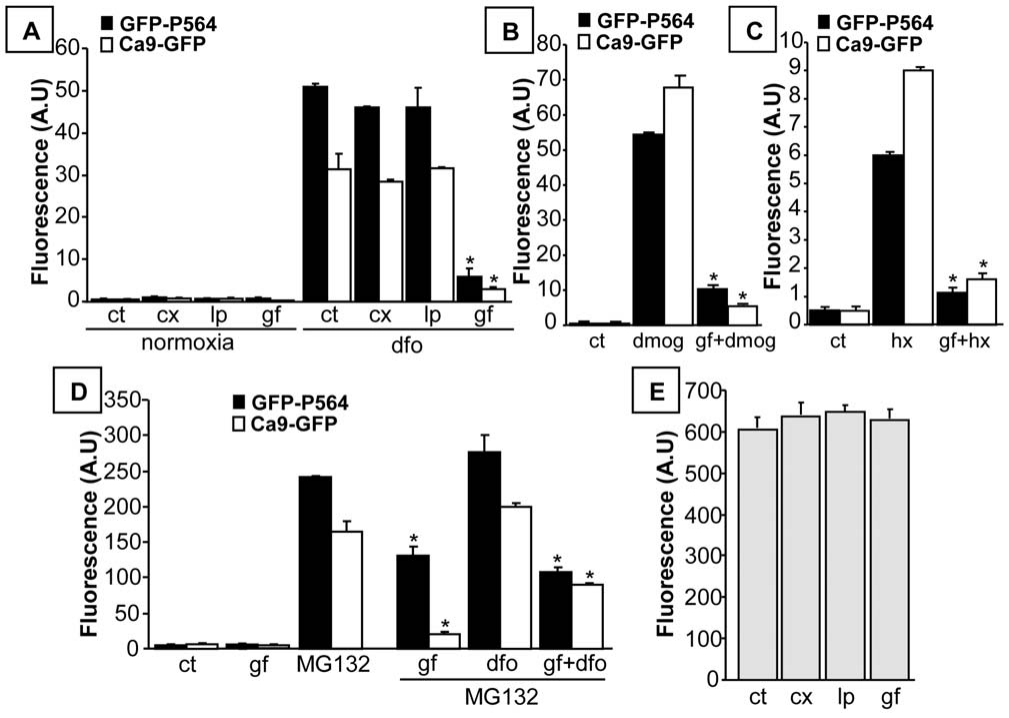
Effect of EGFR inhibitors on hypoxia-induced responses in GFP-P564 and Ca9-GFP cells. Cells were treated with 75 µg/mL cetuximab (cx), 10 µg/mL lapatinib (lp) or 10 µg/mL gefitinib (gf) under hypoxic conditions and stimulated with 200 µM DFO, 5 µM MG132 or 1 mM DMOG or exposed to 1% O_2_ (hx) for 24 H. Fluorescence intensity was assessed by flow cytometry. As controls (ct), cells were incubated under normoxic conditions and left untreated. (A) Cetuximab and lapatinib did not affect the induction of fluorescence by DFO, whereas gefitinib suppressed it in both models. (B-C) Gefitinib also inhibited the induction of fluorescence in GFP-P564 and Ca9-GFP cells in the presence of DMOG and under hypoxia. (D) MG132 is a proteasome inhibitor that induces the stabilization of HIF-1 alpha. Gefitinib diminished the MG132-mediated stabilization of HIF-1 alpha. (E) Cetuximab, lapatinib and gefitinib did not affect the constitutive fluorescence of MDA-MB-231 cells transfected with pEGFP-C1 (CMV-GFP model). Data are shown as the mean ± SEM (n = 3). Statistical differences were assessed using unpaired t-tests; p<0.05 was considered to be significant (* p<0.05).

The overexpression of membrane EGFR, low expression of HER2 receptor and migratory ability were verified in MDA-MB-231 parental cells and the Ca9-GFP and GFP-P564 cell lines using immunocytochemistry and migration assays (data not shown). We examined the effects of EGFR-targeted therapies on the hypoxia-induced stability and activity of HIF complexes. Cetuximab and the two EGFR tyrosine kinase inhibitors (lapatinib and gefitinib) did not affect the signal intensity in either cell model under normoxia. Stimulation with DFO was not influenced by cetuximab or lapatinib treatment, whereas gefitinib strongly reduced the fluorescence by approximately 10-fold (Fig. 4A). The effect of gefitinib was confirmed in the presence of DMOG and under hypoxia (Fig. 4B-C), suggesting that gefitinib reduced the stabilization of HIF alpha isoforms and the activity of HIF dimers through the EGFR signaling pathway. The ability of gefitinib to inhibit the reporter signal was also partially observed in the presence of the proteasome inhibitor MG132, either alone or when combined with DFO (Fig. 4D). This inhibitory effect was more pronounced in Ca9-GFP cells than in GFP-P564 cells.

**Figure 5:**
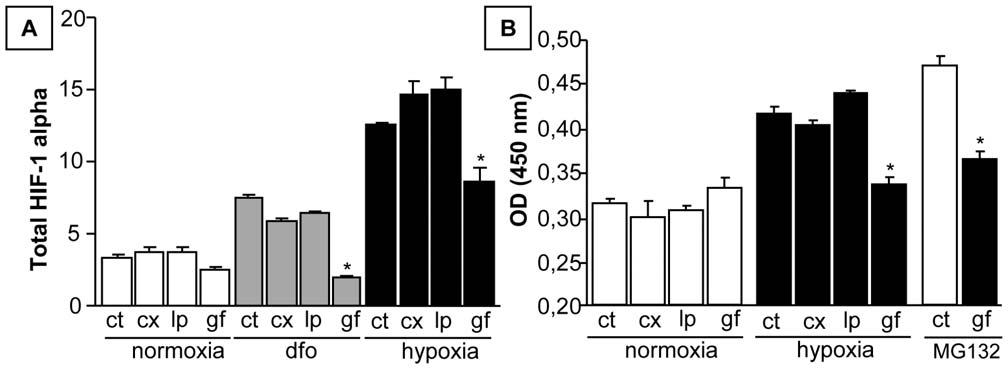
Effect of EGFR inhibitors on HIF-1 alpha in MDA-MB-231 cells under hypoxic conditions. Cells were treated with 75 µg/mL cetuximab (cx), 10 µg/mL lapatinib (lp) or 10 µg/mL gefitinib (gf) under normoxia or hypoxia and stimulated with DFO or MG132 for 24 H. Controls (ct) were left untreated. (A) Total HIF-1 alpha was detected with a whole-cell ELISA assay. Only gefitinib prevented HIF-1 alpha induction by DFO or hypoxia. (B) HIF-1 alpha levels were quantified in nuclear extracts with a DNA-binding assay. Gefitinib reduced the HIF-1 alpha nuclear accumulation induced by hypoxia and MG132. The results are from three independent experiments, and the bars represent the SEM. Student's t-tests were performed to compare the drug responses according to the hypoxia conditions (* p<0.05).

None of the three therapies affected fluorescence in the CMV-GFP cell model that constitutively expressed EGFP, indicating the absence of an interaction between the compounds and the chromophore. Moreover, the construct specificities of the Ca9-GFP and GFP-P564 models were established that way (Fig. 4E). To confirm the observed variation detected by the fluorescent reporters in response to drug treatment under hypoxia, HIF-1 alpha levels were quantified by ELISA in MDA-MB-231 cells. Treatment with cetuximab and lapatinib did not produce any change in HIF-1 alpha expression under normoxia or hypoxic conditions, whereas gefitinib significantly reduced HIF-1 alpha induction in response to DFO and hypoxia (Fig. 5A). DNA-binding assays were also assessed under hypoxia and in the presence of MG132. HIF-1 alpha bound to a consensus HRE was detected using a specific antibody. DNA-binding activity mediated by hypoxia and MG132 only decreased in response to gefitinib treatment (Fig. 5B). These results demonstrated that the hypoxia-induced stabilization and transcriptional activity of HIF-1 could be reversed through EGFR inhibition by selected EGFR-targeted therapies.

### Differential effects of EGFR-targeted therapies on the viability and migratory response of MDA-MB-231 cells

**Figure 6:**
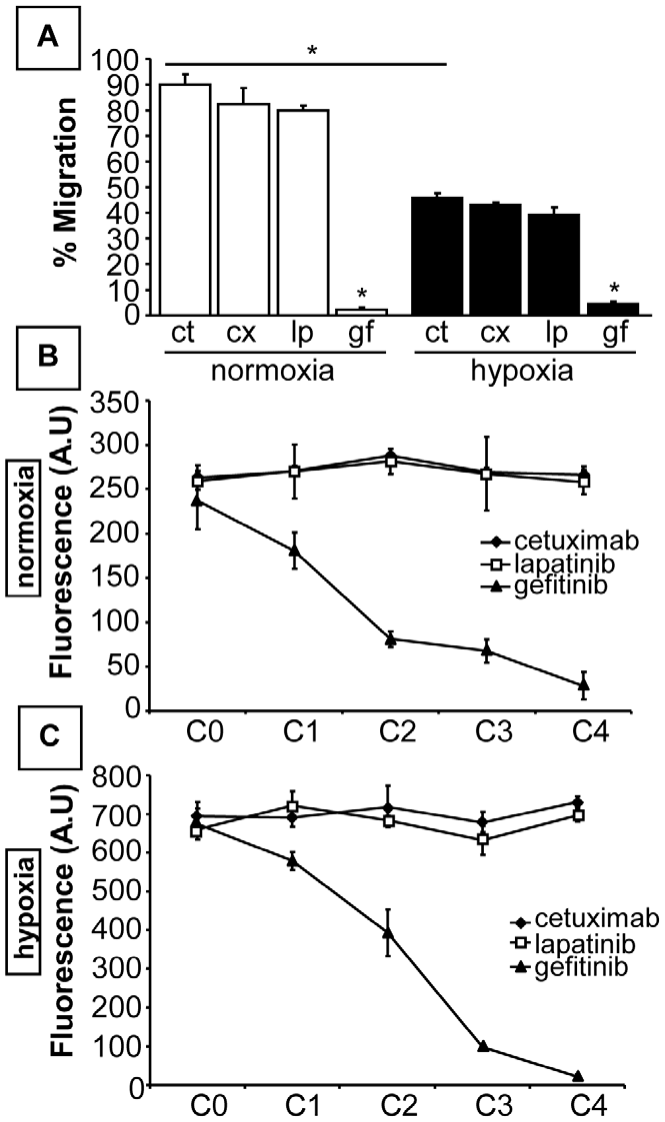
Effect of anti-EGFR-targeted therapies on MDA-MB-231 cell migration and viability. (A) Cells were treated with 75 µg/mL cetuximab (cx), 10 µg/mL lapatinib (lp) or 10 µg/mL gefitinib (gf) and allowed to migrate through inserts (8 µm) under normoxia and hypoxia (1% O2) for 24 H. Cell migration is expressed as the percentage of unmigrated cells. Only gefitinib inhibited MDA-MB-231 cell migration under both conditions. (B) Cells were treated with increasing concentrations of cetuximab (C0:0, C1:25, C2:75, C3:100, C4:150 µg/mL), lapatinib and gefitinib (C0:0, C1:3, C2:10, C3:20, C4:40 µg/mL) under normoxic conditions. Viability was assessed using green calcein-AM labeling and fluorometry (ex: 490/em: 520 nm). Cetuximab and lapatinib did not affect cell viability, whereas gefitinib induced mortality in a dose-dependent manner. (C) A viability assay was performed in a 96-well plate by measuring drug effects under hypoxia in a CMV-GFP cell model that constitutively expressed EGFP. The fluorescence of each well was measured by fluorometry (ex: 488/em: 507 nm), and the signal intensity was proportional to the number of viable cells. Gefitinib strongly induced mortality. The results are representative of at least three independent experiments. Statistical significance was determined by unpaired t-test between treated cells and controls (* p<0.05).

We showed that hypoxia-induced HIF stabilization and activity decreased after gefitinib treatment. Next, we evaluated the migratory response to EGFR inhibitors. Boyden chamber assays or transwell assays using 8-µm filters were assessed under normoxic and hypoxic conditions for 24 H. Under normoxia, 90±9% of untreated cells migrated, whereas only 46±4% migrated under hypoxia. Neither cetuximab nor lapatinib affected cell motility in either condition. In contrast, gefitinib abolished MDA-MB-231 cell migration, even when viability was taken into account. The migratory response was reduced from 90±9% to 2±2% under normoxia and from 46±4% to 5±2% when the cells were exposed to hypoxia (Fig. 6A). We also investigated the sensitivity of the cells to treatment with EGFR inhibitors using calcein-AM staining for viable cells under normoxia. Exposure to increasing concentration of cetuximab and lapatinib did not alter cell viability, whereas gefitinib dramatically induced cell mortality in a dose-dependent manner (IC50 = 14±2.1 µg/mL) (Fig. 6B). Similar results were obtained with the CMV-GFP cell line under normoxia (data not shown) and under hypoxia (Fig. 6C).

## Discussion

TNBC frequently overexpresses EGFR, and this feature represents a promising potential therapeutic target. The EGFR pathway can be targeted by i) an anti-EGFR monoclonal antibody that binds to the extracellular domain of EGFR, such as cetuximab, ii) tyrosine kinase inhibition at the intracellular level, either by a specific inhibitor, such as by gefitinib, which blocks the tyrosine kinase activity of the EGFR intracellular domain, or by an inhibitor with dual specificity, such as lapatinib, a HER2 and EGFR tyrosine kinase inhibitor. Both classes of therapy inhibit the signal transduction of EGFR and are approved for many advanced cancers. However, these therapies benefit only a small proportion of patients. For non-small-cell lung cancer, the population approved for gefitinib is defined by the presence of specific EGFR mutations [29], whereas for colorectal cancer, patients with KRAS mutations are excluded from treatment by cetuximab [30]. Even in those selected population more than half of the patients will not benefit from treatment or will develop resistance. EGFR mutations and KRAS mutations either do not occur or are exceptional in breast cancer. For breast cancer, lapatinib is indicated for advanced HER2 breast cancer. A potential mechanism of resistance to cancer treatment is linked to the hypoxic tumor microenvironment. Cellular adaptations to hypoxia are mainly mediated by HIF-1 alpha accumulation. In breast cancer, the overexpression of HIF-1 alpha is associated with increased mortality [31]. In addition to tumor hypoxia, genetic alterations also can increase HIF-1 alpha levels. Indeed, it has been demonstrated that gain-of-function mutations in oncogenes such as HER2 or EGFR and loss-of-function mutations in tumor-suppressor genes such as VHL or PTEN induce HIF-1 activity [28], [32], [33]. Activation of the PI3K/Akt pathway stimulates HIF-1 alpha protein synthesis through EGFR signaling [27], [34], and several studies have shown that EGFR inhibitors decrease HIF-1 alpha expression in various tumor cell lines [35]. HIF-1 targets genes involved in drug resistance and processes such as extracellular matrix metabolism and cell adhesion, leading to invasion and metastasis. However, the exact mechanism that links HIF-1 to these processes has not yet been elucidated. Furthermore, it seems that this mechanism differs depending upon the cell type considered.

Although basal-like TNBC cell lines express EGF receptor, resistance to anti-EGFR therapies has also been described [5]. To explain this phenomenon, we established two hypoxia-inducible expression systems based on the triple-negative MDA-MB-231 cell line. The GFP-P564 cell model reports the post-translational stability of HIF-1 alpha. The peptide fused in frame with EGFP in this cell model contains HIF-1 alpha amino acids 548 to 583. Hydroxylation of Pro564 has been identified as essential for HIF-1 alpha binding to pVHL, leading to ubiquitin-mediated proteasomal degradation [21], [23]. Furthermore, the interaction between pVHL and HIF-1 alpha has been shown to be dependent on a 27-residue segment comprising residues 549–575 that are highly conserved between different HIF alpha isoforms and also between HIF-1 alpha homologs from human and nonhuman species [36]. Ca9-GFP cells report the transcriptional activity of HIFs through an EGFP reporter under the control of the *Ca9* promoter. The expression of Ca9 is strongly induced by hypoxia in many human cancers [37]. The organization of the *Ca9* promoter with the HRE immediately upstream of the transcription start enables tight regulation by HIF-1 and suggests that Ca9 would be an excellent hypoxic marker in cancers [38], [39]. The native promoter of the *Ca9* gene represents a fine sensor for the transcriptional activation of HIF because the HRE motif plays a crucial role in gene regulation [39], [40]. Moreover, it has been shown that *Ca9* is also regulated in an oxygen-independent manner through the PI3K pathway [41]. Thus, these cell models allowed the potential effects of cetuximab, lapatinib and gefitinib on responses to hypoxia to be assessed. Cetuximab and lapatinib had no effect on hypoxia-induced fluorescence signals in either cell model and did not affect HIF-1 alpha expression in MDA-MB-231 cells. In contrast, gefitinib treatment downregulated HIF-1 alpha and sharply decreased the reporter signal intensity under hypoxia and in the presence of DFO or DMOG. We also observed this effect of gefitinib when the ubiquitin-proteasome pathway was simultaneously blocked by a proteasome inhibitor (MG132) and a PHD inhibitor (DFO). These results suggest that this repression is due, in part, to a mechanism independent of the PHD/VHL/proteasome mechanism. Repression of HIF activity, as seen in Ca9-GFP cells, is consistent with an enhancement of HIF-1 alpha mRNA translation due to AKT/mTOR activation, leading to increased levels of HIF proteins [26], [27], [28]. However, this does not explain the results observed in the GFP-P564 cells.

A differential effect of the EGFR inhibitors on the viability and migration of the TNBC cell line was demonstrated, with these cells being resistant to cetuximab and lapatinib and sensitive to gefitinib at high concentrations. Viability data for cetuximab and gefitinib were consistent with previous observations made using a proliferation assay in the same breast cell line [6]. These differences were correlated with the modulation of HIF expression, stability, DNA-binding capacity and activation under hypoxia. The ability to reduce the hypoxia–induced expression of HIF-1 alpha would be necessary for a positive response of TNBC cells to EGFR-targeted therapy. Several studies have demonstrated that cellular sensitivity to cetuximab and gefitinib required HIF-1 alpha to be downregulated [42], [43], [44]. It has already been suggested that HIF-1 alpha could be a novel response marker for EGFR inhibitors in cancer cell lines that overexpressed EGFR [43]. We also reported here that hypoxia reduced the ability of MDA-MB-231 cells to migrate, regardless of their endogenous HIF-1 alpha status, whereas hypoxia in tumors has been shown to promote invasion and metastasis [45]. Our results were consistent with the observation that MDA-MB-231 cell migration varied depending on the oxygen concentration. Exposure to 1 or 2% O_2_ could reduce MDA-MB-231 cell migration, whereas 5% O_2_ strongly enhanced cell migration [46]. We demonstrated that only gefitinib inhibited cell migration under normoxic and hypoxic conditions. This effect was correlated with the downregulation of hypoxia-induced HIF-1 alpha. Fujiwara et al. reported that downregulation of HIF-1 alpha expression could reduce the migration and invasion of some cancer cell lines *in vitro* [47]. Recently, Li et al. examined the migration of a HIF-1 alpha knockout cell line and reported an inhibition of cell motility compared with wild type cells [48].

To our knowledge, this is the first report describing the differential effects of three EGFR inhibitors on the viability and migration of a TNBC cell line. Downregulation of HIF-1 alpha through the EGFR signaling pathway appeared to be necessary for inducing a positive response to EGFR-targeted therapies, although this may not be sufficient. These results suggest that the activity of HIF-1 alpha in response to EGFR inhibitors will be of interest in preclinical and clinical studies.

## Materials and Methods

### Cell culture and hypoxic conditions

Human MDA-MB-231 metastatic breast carcinoma cells were obtained from the American Type Culture Collection (Manassas, VA, USA). These cells are HER2 negative, estrogen- and progesterone-receptor negative, and overexpress EGFR. Cells were maintained in monolayer culture in a 5% CO_2_ incubator at 37°C in RPMI 1640 medium (Invitrogen Life Technologies, Carlsbad, CA, USA) supplemented with 10% fetal bovine serum, 2 mM L-glutamine and 20 µg/mL gentamicin. To establish hypoxic conditions, cells were plated in 12-well tissue culture plates and placed in a hypoxic chamber (O2sensBox, Adelbio, Clermont-Ferrand, France) flushed with a 1%O_2_/5%CO_2_/94%N_2_ gas mixture for 24 H at 37°C. Alternatively, cells were treated with hypoxia-mimetic compounds for 24 H. Cobalt chloride (CoCl_2_), dimethyloxalylglycine (DMOG) and the iron chelator desferrioxamine (DFO) mimic the effects of hypoxia by inducing HIF-1 alpha, and MG132 is a proteasome inhibitor. CoCl_2_, DMOG, MG132 and DFO were obtained from Sigma-Aldrich (St Louis, MO, USA). With the exception of those used for the dose-response experiments, cells were treated with 200 µM CoCl_2_, 200 µM DFO or 1 mM DMOG for 24 H.

### Drug preparation and treatment

Lapatinib and gefitinib were purchased from LC Laboratories (Woburn, MA, USA). They were dissolved in PBS and stored at −20°C. Cetuximab was supplied by Merck Pharma (Darmstadt, Germany). Cells were treated with increasing concentrations of these drugs under hypoxic and normoxic conditions for 24 H.

### Measurement of HIF-1 alpha protein by ELISA

MDA-MB-231 cells were plated in 96-well plates (10 000 cells per well) and treated with cetuximab (75 µg/mL), lapatinib and gefitinib (10 µg/mL) for 24 H under normoxic and hypoxic conditions (DFO and hypoxia). Conditioned medium was then collected and the cells were fixed and permeabilized with 4% formaldehyde. HIF-1 alpha levels were determined from whole-cell lysates using a human total-HIF-1 alpha Cell-Based ELISA kit according to the manufacturer's protocol (R&D Systems, Minneapolis, MN, USA). Briefly, cells were simultaneously incubated with two primary antibodies, anti-HIF-1 alpha and anti-cytochrome C (normalization antibody). Two labeled secondary antibodies and two fluorogenic substrates were used for detection. Total HIF-1 alpha fluorescence (ex: 540 nm/em: 600 nm) was normalized to the fluorescence of cytochrome C (ex: 360 nm/em: 450 nm).

### HIF-1 alpha DNA-binding assay

MDA-MB-231 cells were treated with cetuximab, lapatinib and gefitinib, as indicated previously, under normoxia and hypoxia. An assay with gefitinib was also performed in the presence of MG132 (5 µg/mL). HIF-1 alpha binding was assessed using Cayman's HIF-1 alpha transcription factor assay (Cayman Chemicals, Ann Arbor, MI, USA). Briefly, a double-stranded oligonucleotide that contained a consensus HIF-1 alpha binding site was immobilized in each well of a 96-well plate and incubated with nuclear extracts (50 µg/well) for 30 minutes at room temperature. The plate was washed and incubated with a primary anti-HIF-1 alpha antibody. A secondary antibody conjugated with horseradish peroxidase (HRP) was used for detection. The DNA-binding activity of HIF-1 alpha was measured by absorbance and was expressed as the optical density at 450 nm (OD450). This kit used a positive control for HIF-1 alpha activation and a specific competitor dsDNA for HIF-1 binding to the plate to monitor the specificity of the assay.

### Western blot analysis

Nuclear extracts were obtained with a nuclear extraction kit (Cayman chemicals), and protein concentrations were determined using a bicinchoninic acid assay kit (Bio-Rad Laboratories, Hercules, CA, USA). Each sample (50 µg) was separated by electrophoresis on a 12% SDS-polyacrylamide gel, and the proteins were transferred onto a nitrocellulose membrane (Bio-Rad Laboratories). The membrane was blocked in 5% milk in Tris-buffered saline with 0.1% Tween (TBST) at 4°C for one hour and incubated under mild agitation with a monoclonal anti-HIF-1 alpha antibody (Novus Biologicals, Littleton, CO, USA) diluted 1∶1500 at 4°C overnight. Membranes were washed three times in TBST and incubated for one hour with an HRP-conjugated secondary antibody (diluted 1∶2000), and detection was performed using an enhanced chemiluminescence detection system (GE Healthcare, Piscataway, NJ, USA).

### DNA constructs

The *GFP-P564* DNA construct encodes a peptide containing the Pro564 residue of human HIF-1 alpha (Genbank accession number: U22431) fused in-frame to the 3′ end of the enhanced green fluorescent protein (EGFP) in a pEGFP-C1 vector (Clontech, Mountain View, CA, USA). This peptide is encoded by a 135-bp DNA fragment corresponding to amino acids 543-587 of human HIF-1 alpha. PEGFP-C1 is a plasmid coding for EGFP driven by a human cytomegalovirus promoter (CMV). The *Ca9-GFP* DNA construct was obtained by integrating the sequence between −522 and +9 from the transcriptional start site of the *carbonic anhydrase 9* (Ca9) promoter (Genbank accession number: NM_001216) into a pEGFP-1 vector (Clontech).

### Stable transfection

The GFP-P564 and Ca9-GFP cell lines were obtained by the stable transfection of MDA-MB-231 cells with the molecular constructs described above using the Fugene-6 reagent kit (Roche, Mannheim, Germany). The medium was removed 24 hours after transfection, and the cells were supplemented with complete media containing 600 µg/ml of G418 (Invitrogen) for selection for 3 weeks; the cells were subsequently grown in the presence of 100 µg/mL G418. Stable GFP-P564 and Ca9-GFP cells were cloned by limiting dilution, and reporter expression was determined by flow cytometry (Beckman Coulter, Miami, FL, USA) in cells exposed to 200 µM DFO and 1 mM DMOG for 24 H, in order to select hypoxia-inducible clones. EGFP has excitation and emission maxima at 488 nm and 507 nm, respectively. A clone transfected with an empty pEGFP-C1 vector that constitutively expressed EGFP (CMV-GFP) was used as control for the GFP-P564 and Ca9-GFP experiments. Direct sequencing was used to verify the authenticity of each construct before and after the transfection assay.

### Cell migration assay

MDA-MB-231 cell migration was assessed using a modified Boyden chamber method with a 24-well plate. The filter was a polyethylene terephthalate membrane with 8-μm pores (Millipore, Billerica, MA, USA). Briefly, cell suspensions were seeded into the upper chamber of the inserts (10 000 cells per well), and the inserts were placed into a well that contained conditioned media. Cells were treated with 75 µg/mL cetuximab, 10 µg/mL lapatinib and 10 µg/mL gefitinib under normoxia or hypoxia for 24 H. Non-migrating cells were removed by gentle scraping and were counted using trypan blue (Invitrogen). The migratory responses to EGFR inhibitors were quantified by the percentage of cells that migrated. Triplicate wells were analyzed in each assay, and the assay was repeated at least three times.

### Cell viability assay

A fluorogenic esterase substrate (Calcein-AM, Sigma-Aldrich) was used to investigate cell viability under hypoxia in response to EGFR inhibitors. Cells were seeded in black-walled microplates and treated with 5 mM of Calcein-AM for one hour at 37°C. Viability was determined by measuring fluorescence using a 490 nm excitation filter and a 520 nm emission filter. The fluorescence intensity was assumed to be proportional to the number of viable cells. The effect of the drugs was also analyzed under normoxia with a CMV-GFP cell line that constitutively expressed EGFP. Cells were seeded on a 96-well plate, and at 80–90% confluence, the cells were treated with increasing concentrations of EGFR inhibitors. After washing with PBS, the signal intensity of each well was quantified by fluorometry (ex: 488 nm/em: 507 nm).

### Statistical Analysis

All experiments were repeated at least 3 times. All data are presented as the mean ± SEM. An unpaired Student's t-test was used to compare the treated and control groups. A probability value p<0.05 was considered significant.
